# Kinetics of diffusion-controlled enzymatic reactions with charged substrates

**DOI:** 10.1186/1757-5036-3-1

**Published:** 2010-01-18

**Authors:** Benzhuo Lu, J Andrew McCammon

**Affiliations:** 1 State Key Laboratory of Scientific/Engineering Computing, Institute of Computational Mathematics and Scientific/Engineering Computing, Academy of Mathematics and Systems Science, Chinese Academy of Sciences, Beijing 100190, China; 2 Department of Chemistry and Biochemistry, Center for Theoretical Biological Physics, Department of Pharmacology, Howard Hughes Medical Institute, University of California at San Diego, La Jolla, CA, 92093-0365, USA

## Abstract

The Debye-Hückel limiting law (DHL) has often been used to estimate rate constants of diffusion-controlled reactions under different ionic strengths. Two main approximations are adopted in DHL: one is that the solution of the linearized Poisson-Boltzmann equation for a spherical cavity is used to estimate the excess electrostatic free energy of a solution; the other is that details of electrostatic interactions of the solutes are neglected. This makes DHL applicable only at low ionic strengths and dilute solutions (very low substrate/solute concentrations). We show in this work that through numerical solution of the Poisson-Nernst-Planck equations, diffusion-reaction processes can be studied at a variety of conditions including realistically concentrated solutions, high ionic strength, and certainly with non-equilibrium charge distributions. Reaction rate coefficients for the acetylcholine-acetylcholinesterase system are predicted to strongly depend on both ionic strength and substrate concentration. In particular, they increase considerably with increase of substrate concentrations at a fixed ionic strength, which is open to experimental testing. This phenomenon is also verified on a simple model, and is expected to be general for electrostatically attracting enzyme-substrate systems.

**PACS Codes**: 82.45.Tv, 87.15.Vv

**MSC Codes**: 92C30

## 1 Background

Electrostatically steered diffusion-reaction processes exist widely in chemistry and biochemistry [1,2]. Ionic screening effects were first described by using the well-known Debye-Hückel limiting law (DHL) [3]. The DHL implemented within transition state theory [4] is still often used to estimate the kinetics of enzyme-substrate reactions. E.g., the dependence of the rate constant on ionic strength for the diffusion-controlled reaction of acetylcholine (charge = +1) catalyzed by acetylcholinesterase can be described approximately by [5]:

where *k*_on_,  and  are second-order association rate constants at the specified ionic strength *I*, zero ionic strength, and infinite ionic strength, respectively. *z*_*E *_and *z*_*I *_are the charges of the enzyme and substrate involved in the interaction. The DHL says that the rate constant (in an electrostatically-steered process) decays exponentially with the increase of the square root of ionic strength, as is observed under some conditions [5-8]. However, because the DHL is based on an excess free energy described by the linearized Poisson-Boltzmann model of an ionic solution, it is assumed that the ionic species involved obey a Boltzmann distribution, i.e. are in an equilibrium state. The contributions of solute-solute interactions to the excess free energy are ignored in the theory. Moreover, in diffusion-influenced reactions, the substrate distribution is not in an equilibrium state. Therefore, the DHL only applies for low ionic strengths, and very dilute substrate concentrations. However, in real biological systems, the substrate concentration can be quite high; e.g., the acetylcholine concentration can reach about 300 mM when released from vesicles in synapses [9].

The finite concentration effect was recently studied using Brownian dynamics simulation [10], and later theoretical work was done for the condition of weak substrate-substrate interaction or low substrate density [11]. Both works are idealized studies for spherical models of enzymes. Here, we use newer methods to compute the reaction rates for more realistic models at diverse ionic and substrate concentrations. We show that the results display a more complicated dependence of the reaction coefficient upon the ionic strength and the substrate concentrations.

## 2 Methods

We use a continuum model to simulate the electrodiffusion processes. The theoretical background is introduced in [8]. In the present work, the Poisson-Nernst-Planck equations are solved to determine the substrate flux driven by the full electrostatic field, including the influence of substrate itself, salt ions (e.g., Na^+ ^and Cl^-^), and the atomic charges of the enzyme. Usually, we use three NP equations to describe the diffusion of three mobile species (counterion, coion, and substrate) respectively in the PNP model:

where *p*^*i*^(*r*) is the density distribution function of the diffusing particles of the *i*-th species with diffusion coefficient *D*^*i *^and charge *q*^*i*^, ρ^*f *^is the fixed atomic charge distribution on the enzyme, β is the inverse Boltzmann energy, ε is the dielectric coefficient, ϕ is the electrostatic potential determined by the Poisson equation. The flux is

The reaction between the enzyme and the diffusing substrate is modeled by an absorbing boundary condition on a reactive site represented by a molecular surface patch. It is worth noting that this treatment is due to the fact that acetylcholinesterase is considered a fast enzyme. But in the context of high concentration of acetylcholine, ca. 500 mM, the simultaneously absorbing boundary condition may not be proper due to limited diffusion speed. In such case, a more complicated boundary condition such as Robin boundary condition, or inclusion of coupled ordinary partial differential equations can serve as a better description of the reaction event. The implementation of these considerations would be a future direction. For consistency and convenience in the setup of the computational model, a same absorbing boundary condition is used in this work. The reaction rate *v *is determined by integrating the flux *J *of substrate particles at the reactive site, i.e., *v *= ∫*J*·*ndS*, and the rate coefficient (for steady-state) *k *is defined as *k *= , where *C *is the bulk substrate concentration. We note that the DHL can be well reproduced in the continuum model when the substrate density is not coupled into the full electric field [7,8,12]).

Calculations of steady-state rate coefficients are performed for the enzyme catalyzed degradation of acetylcholine (ACh), which is an electrostatically steered diffusion-controlled reaction [5]. ACh carries one positive unit of charge, and the enzyme, acetylcholinesterase (AChE), carries a fixed charge of -7.61e. The partial atomic charge and van der Waals radii are taken from the AMBER force-field [13]. In a synapse, there is a certain background concentration of "spectator" ions (ca. 150 mM ionic strength), and then additional ions (the ACh and its counterions) are released (initially ca. 300 mM ionic strength) as a vesicle opens. The actual system is of course non-steady, but for initial steady-state calculations in this work, we focus on how the substrate concentration affects the reaction rate coefficients. Suppose that there are only monovalent ions in the salt, that *C*_+ _and *C*_- _are the total bulk concentrations of cation and anion respectively, and that *C*_subs _is the bulk concentration of substrate ACh. These bulk concentrations are used as the outer boundary conditions of the diffusion domain in solving the PNP equations [8]. Therefore, to make a closer connection with physiology, it is reasonable to consider a neutrality condition of the bulk solution in this work as *C*_+ _+ *C*_subs _- *C*_- _= 0. As a comparison, the condition *C*_+ _- *C*_- _= 0 will lead to quite different results, which will be addressed later.

## 3 Results

**Figure 1:**
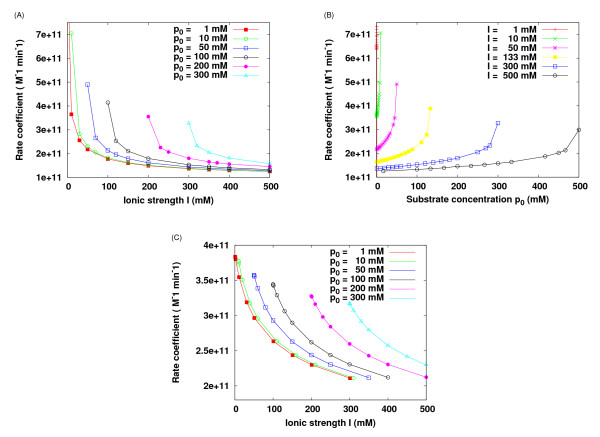
**Reaction rate affected by both ionic strength and substrate concentration**. Reaction rate coefficients for (a-b) ACh-AChE reaction system, (c) an absorbing unit sphere model system. *p*_0 _is bulk substrate concentration; *I *is total ionic strength (spectator ions plus substrate).

The reaction rate coefficient is shown as a function of ionic strength (= "spectator" + bulk substrate) for different prescribed substrate concentrations in Figure 1(a) and as a function of bulk substrate concentration for different prescribed ionic strengths in Figure 1(b). The results show that the reaction rate coefficients strongly depend on both ionic strength and substrate concentration. At very low substrate concentration, e.g., 1 mM or less, the results show asymptotic agreement with the DHL (see red line in Figure 1(a)). However, at moderate concentrations of the substrate, the curves are shifted. A general trend is observed: the rate coefficient increases as the bulk/distant concentration of substrate increases for a fixed overall ionic strength. For instance, for a fixed ionic strength of 300 mM (*C*_+ _+ *C*_subs _= 300 mM), the rate coefficient is 1.36 × 10^11 ^M^-1^min^-1 ^for *C*_subs _= 1 mM and is increased to 3.28 × 10^11 ^M^-1^min^-1 ^for *C*_subs _= 300 mM. The physical origins of the observed behavior can be explained as follows. If substrate concentration is not considered, as in most previous work based on the DHL, the concentration of the counter ion of the enzyme, i.e., *C*_+ _here, is equal to the concentration of the coion, i.e., *C*_+ _= *C*_-_. The counter ions are attracted and concentrated around the negatively charged active site, which serves to screen the Coulomb interaction between ACh molecules and AChE, hence slowing the association. When *C*_subs _is considered in the PNP model, to maintain the same ionic strength, *C*_+ _needs to be reduced by *C*_subs _compared with that in the familiar Debye-Hückel theory. This leads to a thinner counter-ion atmosphere around the active site, and it can not be compensated by the additional substrate (ACh) density that is relatively low due to reactant depletion that results from the absorbing boundary condition. In other words, in the resulting non-equilibrium state, the sum of counter-ion density and ACh density near the active site is lower than that obtained with the Boltzmann distribution for a +1e particle. The consequences are a reduced overall screening effect and thereby an enhanced reaction rate.

The ionic atmosphere always screens the electrostatic interactions, and hence reduces the rate coefficients. At very high ionic strength, due to strong ionic screening effects, the electrostatic interactions become very weak. This is close to the pure diffusion case, and all the rate constants for different substrate concentrations are close to the pure diffusion-reaction rate constant.

## 4 Discussion

The phenomena observed above are expected to be general for attractive substrate-enzyme systems, which can be illustrated with an idealized sphere model. Figure 1(c) shows the results using an absorbing unit sphere with one positive charge +e located in the center, and assuming that a substrate molecule brings a negative charge -e. We note in passing that the results in Figure 1 suggest that, for each fixed substrate concentration, it may be possible to fit the rate coefficients to a DHL-like curve again.

As a comparison, if we use an aforementioned neutrality condition *C*_+ _- *C*_- _= 0 and the substrate concentration is not counted into ionic strength as was done in our former work [12], very different trends will be found. The present results provide a more complete and realistic of the biophysics of the ACh-AChE system.

However, it is worth pointing out an issue in this model that the reaction products, choline and acetic acid that will ionize and generate acetate and a proton, are also charged species. The distribution and diffuse of these added ionic species will definitely affect the local ionic strength, hence the reaction rate coefficient. Therefore, a more complete treatment of some enzymes that catalyze reactions of charged substrates should include additional species such as charged products of the reaction in the model. But this brings the methodology a new issue that is how to elaborate the current PNP model to include the product diffusion originated from the reactive site, which will lead to some additional lines of work in the future. Therefore, the present work is limited to the case in which product concentrations are small, so that experimental tests of the current model would need to be in the early steady-state regime.

## 5 Conclusions

To summarize, the DHL only applies to very dilute situations. Our numerical results show that for electrostatically steered diffusion-controlled reaction processes, the rate coefficients strongly depend on both ionic strength and substrate concentration. At the same ionic strength, the current model predicts that increasing substrate concentration results in significant increase in rate coefficients for the attractive substrate-enzyme systems in case the product concentration can be ignored (like in the early steady-state regime). We are extending the theory and simulation methods to account for finite product concentrations, which will allow for easier comparison with experiments.
